# Characterization of the Microbiome along the Gastrointestinal Tract of Growing Turkeys

**DOI:** 10.3389/fmicb.2017.01089

**Published:** 2017-06-22

**Authors:** Toby J. Wilkinson, A. A. Cowan, H. E. Vallin, L. A. Onime, Linda B. Oyama, S. J. Cameron, Charlotte Gonot, J. M. Moorby, K. Waddams, V. J. Theobald, D. Leemans, S. Bowra, C. Nixey, Sharon A. Huws

**Affiliations:** ^1^Institute of Biological, Environmental and Rural Sciences, Aberystwyth UniversityAberystwyth, United Kingdom; ^2^Department of Surgery and Cancer, Faculty of Medicine, Imperial College LondonLondon, United Kingdom; ^3^Phytatec (UK) Ltd.–Plas GogerddanAberystwyth, United Kingdom; ^4^British Poultry CouncilLondon, United Kingdom

**Keywords:** turkey, 16S rDNA, microbiome, *Campylobacter*, gastrointestinal tract, small intestine, caecum, large intestine

## Abstract

The turkey microbiome is largely understudied, despite its relationship with bird health and growth, and the prevalence of human pathogens such as *Campylobacter* spp. In this study we investigated the microbiome within the small intestine (SI), caeca (C), large intestine (LI), and cloaca (CL) of turkeys at 6, 10, and 16 weeks of age. Eight turkeys were dissected within each age category and the contents of the SI, C, LI, and CL were harvested. 16S rDNA based QPCR was performed on all samples and samples for the four locations within three birds/age group were sequenced using ion torrent-based sequencing of the 16S rDNA. Sequencing data showed on a genus level, an abundance of *Lactobacillus, Streptococcus*, and *Clostridium XI* (38.2, 28.1, and 13.0% respectively) irrespective of location and age. The caeca exhibited the greatest microbiome diversity throughout the development of the turkey. PICRUSt data predicted an array of bacterial function, with most differences being apparent in the caeca of the turkeys as they matured. QPCR revealed that the caeca within 10 week old birds, contained the most *Campylobacter* spp. Understanding the microbial ecology of the turkey gastrointestinal tract is essential in terms of understanding production efficiency and in order to develop novel strategies for targeting *Campylobacter* spp.

## Introduction

Poultry meat represents the main source of protein for human nutrition with consumption per capita being nearly twice that of red meat (Foley et al., 2011). Globally the US consumes the most poultry meat with the European Union following closely (average 22.2 Kg/capita in 2006 for EU) (Magdelaine et al., 2008). Of this around 17% is attributable to turkey (*Meleagris gallopavo*) consumption, with chickens (*Gallus gallus domesticus*) being the main poultry consumed. The reason for poultry meat's popularity is attributed to leanness and lower price compared to most other meats.

The relationship between the gut microbiome of chickens, to bird health and efficient growth is well-known (Brisbin et al., 2008; Scupham et al., 2008; Yeoman et al., 2012; Danzeisen et al., 2013; Wei et al., 2013). Scupham et al. (2008) also showed that high density turkey production has altered the caecal microbiome of the turkey as compared with their wild counterparts. Recent next generation sequence-based data also shows that the gastrointestinal tract (GI) microbiome of turkeys is reasonably distinct to that found in chickens, with only 16–19% similarity at a species level (Wei et al., 2013, 2016). It is also known that the turkey GI tract microbiome changes during the turkey growth phase (up to 7 weeks) but few studies expand further into maturity (Scupham, 2009; Danzeisen et al., 2015).

The prevalence of the food-poisoning bacterium *Campylobacter* spp. in poultry products is also a cause of major concern in terms of economic impact to the industry and human health (Silva et al., 2011). *Campylobacter* is the main cause of food-poisoning in developed countries, with 70,298 cases reported in the UK in 2011 (DEFRA, 2011). Nonetheless, this estimate is conservative as many cases are not reported (Tam et al., 2012). *Campylobacter* spp. and their effect on bird health is disputed, with many researchers believing that this genus causes minimal detrimental effect on the health of poultry. Nonetheless, once ingested by humans *Campylobacter* spp. cause diarrhea, abdominal pain and nausea which last between 5 and 7 days, with 10% of cases ending in hospitalization and 0.2% in death (MacRitchie et al., 2014; Thibodeau et al., 2015). It is also known that the infective dose required to cause illness in humans is only around 500 colony-forming units (Waag et al., 1999). Campylobacteriois in humans is normally associated with GI tract contamination of the poultry carcass during slaughter (Oakley et al., 2013; MacRitchie et al., 2014), and recent studies illustrate that up to 76% of carcasses in supermarkets have *Campylobacter* contamination at levels capable of causing illness (Skarp et al., 2016). Due to the predominant consumption of chicken, emphasis on understanding the chicken microbiome and specifically developing novel strategies to combat food poisoning linked to *Campylobacter* has focused on these birds, with much less emphasis on turkeys. However, *Campylobacter* spp. also inhabit the GI tract of other birds used for human consumption, including turkeys (Wei et al., 2013; Danzeisen et al., 2015; Skarp et al., 2016). It is assumed that *Campylobacter* spp. reside mainly within the caeca of turkeys akin to the situation in chickens, although few studies have investigated this using recently developed next generation sequencing, particularly early development of the birds to maturity.

The aims of this study were to assess bacterial diversity within the turkey small intestine, caeca, large intestine and cloaca at 6, 10, and 16 (slaughter age) weeks of age using next generation sequencing, coupled with quantitative PCR for detection of thermophilic *Campylobacter* spp. Increased understanding of the turkey microbiome, in particular *Campylobacter* spp., and colonization of the different parts of the turkey GI tract over time, will aid our understanding of turkey health and development of effective control interventions to limit cases of human Campylobacteriosis.

## Materials and methods

### Study design and sample harvesting

All work described using animals was conducted in accordance with the requirements of the UK Animals (Scientific Procedures) Act 1986 and with the approval of the Aberystwyth University Animal Welfare and Ethical Review Body. Turkeys were humanely euthanized using captive bolt by a registered license holder. Twenty-four female turkeys were obtained from a commercial producer at 5 weeks of age. The turkeys were subsequently reared in a broiler unit until 8 were slaughtered at each time point (6, 10, and 16 weeks of age). Birds were fed a turkey grower mash (Table 1) (GLW Feeds Ltd, Loughborough, UK). All birds had constant access to fresh water. After slaughter the gastrointestinal tract of the turkeys were dissected and the contents from the whole small (SI) and large (LI) intestines were taken, whilst sub-samples of caeca (C), and cloacal (CL) material were taken and stored at −20°C for assessment of the bacterial diversity and abundance as detailed below.

**Table 1:** Nutritional components of the turkey feed.

**Ingredient**	**Part (% of fresh feed)**
Wheat	40.17
Braz/Para hipro soya	21.2
Oat-X	20
Wheatfeed meal	8
Monogastric remix	3
Sunflower ext (36)	3
Dical phosphate (18%)	1.48
Liquid lysine 50(T)	0.68
Limestone flour	0.6
DSM-ATL Turkey 2	0.5
Methionine H-A liquid	0.44
Soya oil spray	0.3
Salt	0.23
Liquid fat	0.2
L-Threonine	0.14
Elancoban G200 (E:757)	0.03
Roxazyme G2G liquid 35.7%	0.03
Natuphos 5000 liquid	0.01

### DNA extraction

Genomic DNA was extracted from the turkey intestinal samples (10 mg fresh weight) using the BIO101 FastDNA® SPIN Kit for Soil (Qbiogene, Cambridge, UK) in conjunction with a FastPrep® cell disrupter instrument (Bio101, ThermoSavant, Qbiogene) according to the manufacturer's instructions with the exception that the samples were processed for 3 × 30 s at speed 6.0 in the FastPrep instrument. DNA was quantified and quality-assured using the Epoch microplate spectrophotometer (Biotek, Bedfordshire, UK).

### 16S rDNA ion torrent PGM sequencing

16S rDNA ion torrent sequencing was completed for all GI tract locations for 3 birds within each of the age categories (6, 10, and 16 weeks), resulting in 36 samples being sequenced in total. Only 36 sequences were sampled as this was the maximum that could be sequenced on the ion torrent chip, whilst providing the sequencing depth required. Amplicons of the V1–V2 variable region of the bacterial 16S rDNA gene were generated in triplicate for each of the 36 samples by PCR using the primers 27F (5′AGAGTTTGATCMTGGCTCAG 3′) and 357R (5′ CTCCTACGGGAGGCAGCAG 3′) followed by ion torrent sequencing using adaptors as described by Belanche et al. (2016). All PCR products were initially verified by electrophoretic fractionation on a 1.0% agarose gel for 1 h, 120 V, and 80 MA in 1% TAE (Tris base, acetic acid and EDTA) buffer before pooling of triplicate amplicons. The pooled PCR products (30 μl each sample) were subsequently run on a 2.0% agarose gel for 2 h, 120 V, and 80 MA in 1% TAE buffer before bands were viewed and cut on a dark reader transilluminator (Clare Chemical Research, Colorado, USA). Amplicons were retrieved from cut bands using the Isolate II PCR and Gel Kit (Bioline, London, UK). Purified amplicons were verified and quantified using the Agilent High Sensitivity Assay Kit (Agilent Technologies, California, USA) prior to sequencing using the Ion Torrent PGM sequencer following the Ion PGM Template OT2 400 and Ion PGM Hi-Q Sequencing kits (Life Technologies Ltd, Paisley, UK). These sequences have been submitted to the short read archive in the NCBI database under accession number PRJEB14286.

### Quantitative PCR

Total bacterial 16S rDNA QPCR were carried as described by Huws et al. (2013), Huws et al. (2014a,b), and Huws et al. (2016) for all samples generated (8 birds × 3 ages × 4 locations = 96 samples). *Campylobacter* spp. were detected using a QPCR method developed by Lund et al. (2004), targeting 16S rDNA, and again for all 96 samples. Essentially, the reaction mixture (25 μl) contained 1 × POWER SYBR green PCR Master Mix (Applied Biosystems, Warrington, UK), 10 pmol of each primer (campF2 - 5′-CACGTGCTACAATGGCATAT-3 and campR2 - 5′-GGCTTCATGCTCTCGAGTT-3′), 10 pmol of the probe (campP2 - 5′-FAM-CAGAGAACAATCCGAACTGGGACA-BHQ1-3), and 2 μL of template DNA (*ca*. 20 ng). Amplification for each QPCR involved 50°C for 2 min, 95°C for 10 min, followed by 45 cycles of 95°C for 15 s, followed by annealing at 58°C for 30 s, extension at 72°C for 30 s, with a final cycle of 5 min at 72°C. All samples were run in duplicate. A *Campylobacter* spp. QPCR standard was prepared using DNA extracted from *C. jejuni* NCTC 11322.

### Taxonomy and functional gene prediction

Using the CD-HIT-OTU pipeline (Li et al., 2012) sequences were denoised, low quality sequences, pyrosequencing errors and chimeras were removed, then sequences were clustered into Operational Taxonomic Units (OTU's) at 97% identity. OTU's containing fewer than 10 reads were excluded due to the likelihood of them being a sequencing artifact. OTUs were classified against the Greengenes 16S rRNA gene database (13.5) using MOTHUR (Schloss et al., 2009) and the taxonomy added to the OTU table. Phylogenetic Investigation of Communities by Reconstruction of Unobserved States 165 (PICRUSt) was used to predict the genomic and metabolic potential represented by the microbiota at each GI tract location in the different turkey ages. Using functions within the PICRUSt pipeline this was then normalized and used for metagenome inference of Kyoto Encyclopedia of Genes and Genomes (KEGG) orthologs. The predicted functions (KOs) were then collapsed into hierarchical KEGG pathways using the categorize_by_function step in the PICRUSt pipeline.

### Statistical analysis

Principal component analysis ordination plots of OTU data were constructed using the Phyloseq program for R (McMurdie and Holmes, 2013). Taxonomical tables at phyla and genera (converted to % of total reads) were subjected to analysis of variance (ANOVA) with sample as the fixed effect and blocking by turkey using GenStat (Payne et al., 2007). Interactions between GI location and turkey age were also investigated using ANOVA and phylum and genus level data. Calculations of alpha diversity and beta dispersion were performed using the phyloseq Bioconductor package in R, multivariate ANOVA of bray-curtis distance matrices were assessed by 1,000 permutations and corrected using the Bonferroni method (McMurdie and Holmes, 2013). QPCR data was also subjected to ANOVA with sample as the fixed effect and blocking by turkey using GenStat (Payne et al., 2007). Interactions between GI location and turkey age were also investigated using ANOVA for the QPCR data. Analysis of corrrelation between *Campylobacter* presence/abundance and microbiome composition was carried out at OTU and genus level using the Bioconductor package metagenomeSeq in R (Paulson et al., 2013). Statistical Analysis of Metagenomic Profiles (STAMP) was employed to analyse PICRUSt data. Samples were blocked by age and location, and subjected to ANOVA (multiple groups) with 1,000 permutations, Tukey-Kramer *post-hoc* analysis and corrected for multiple testing using the Bonferroni method. STAMP was further used to produce principle coordinate analysis (PCA) and extended error bar plots based on these analyses.

## Results

### Sequencing data

Post-quality control, we obtained a total of 4,485,560 reads of sequencing, averaging at 90,267reads/sample (Table 2). Average sequence length was 415 bp (Table 2).

**Table 2:** Sequencing information on average across sampling site and age.

**Sequence information**	**Average bp (reads)**
Pre QC: Base pair (bp) count	37,511,950 (4,485,560)
Post QC: bp Count	18,086,244 (90,267)
Post QC: Sequence count	502,396 (10,351)
Post QC: Mean sequence length	415 (27)

### The turkey microbiome along the gastrointestinal tract

**Figure 1:**
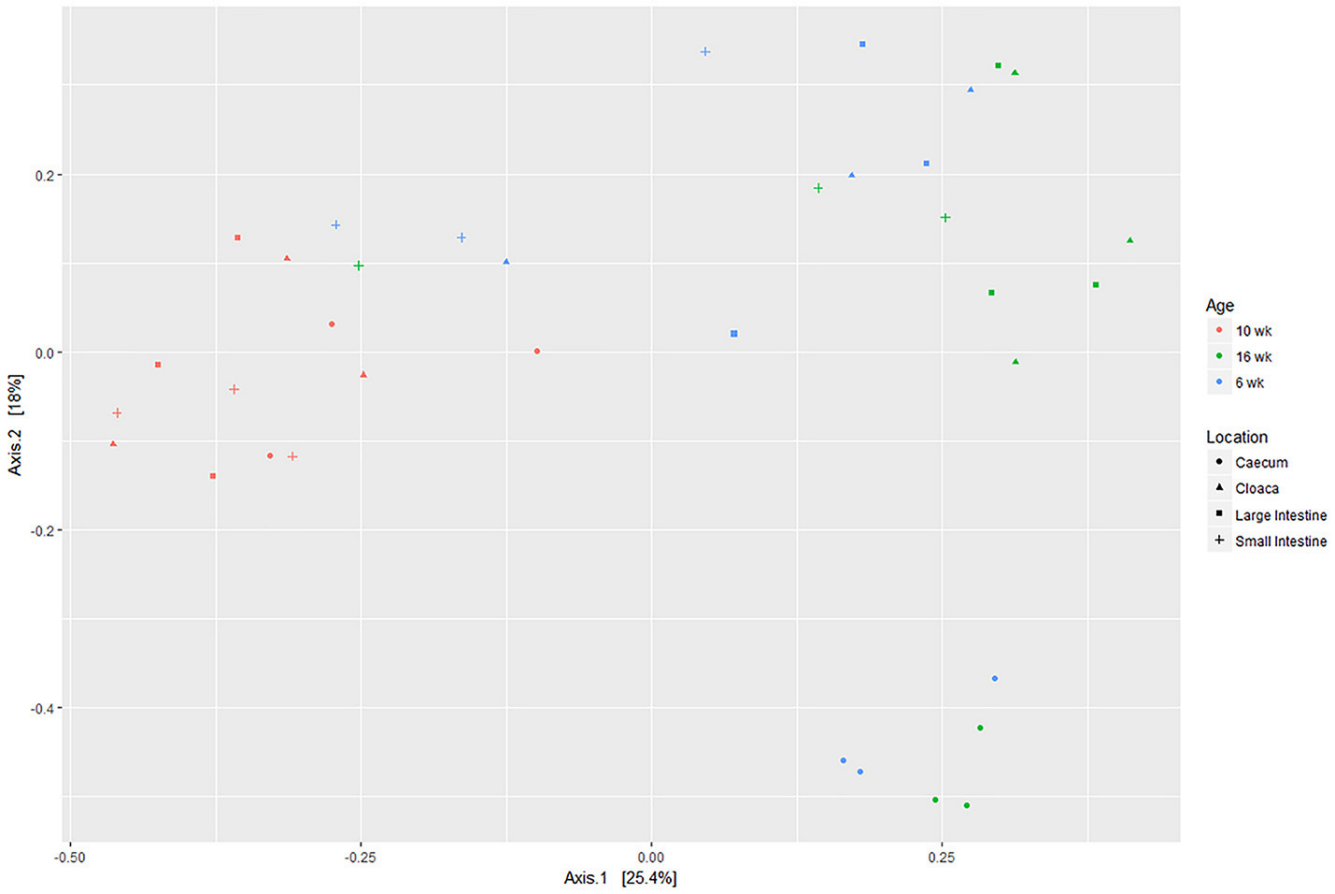
Principal component analysis ordination plots generated using Phyloseq for R based on operational taxonomic units. Wk, week.

**Figure 2:**
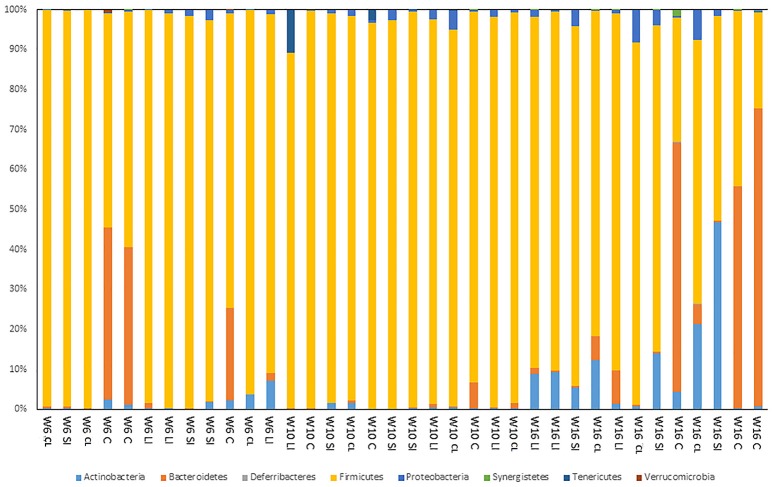
Proportional representation of the turkey gut microbiome on a phylum level across the GI tract of birds at 6, 10, and 16 weeks of age. W, Week; SI, Small intestine; LI, Large intestine; C, Caeca; CL, Cloaca gut microbiome.

The PCA plot based on OTUs showed that the microbiome of 10 week old birds was quite distinct to those of 6 and 16 week old birds irrespective of GI tract location (Figure 1). On a phyla level, and irrespective of GI tract location and bird age, Firmicutes predominated (on average 84.5% of all sequence reads) (Figure 2, Table 3). Bacteroides, Actinobacteria, and Proteobacteria were the next predominating phyla after Firmicutes with an average abundance of 9.30, 4.11, and 1.48% of total reads respectively (Figure 2, Table 3). On a phylum level, the main differences seen were that Bacteroidetes were more abundant in the caeca and firmicutes less abundant compared with other GI tract locations (Figure 2, Table 3). In terms of age, Bacteroidetes was lower in the GI tract of 10 week old birds, whilst Firmictues were higher compared with turkeys of 6 and 16 weeks of age (Figure 2, Table 3). Significant interactions between age and GI tract locations were also evident for the Bacteroidetes and Firmicutes (Table 3).

**Table 3:** Bacterial phyla present within the small intestine, caecum, large intestine, and cloaca of mature turkeys.

**Bacterial phylum**	**Sample location**				**Age**			**SED**		***P***		
	**SI**	**LI**	**C**	**F**	**6**	**10**	**16**	**Age**	**Sample**	**Age**	**Sample**	Age^*^Sample
Actinobacteria	7.80	3.10	1.30	4.50	1.60^a^	0.40^a^	10.40^b^	3.12	3.60	0.006	0.334	NS
Bacteroidetes	0.20^a^	1.60^a^	33.90^b^	1.60^a^	9.20^ab^	0.80^a^	17.90^b^	5.02	5.80	0.008	<0.001	<0.001
Deferribacteres	0.00	0.00	0.05	0.00	0.00	0.00	0.04	0.04	0.05	NS	NS	NS
Firmicutes	90.00^b^	93.00^b^	63.80^a^	91.30^b^	88.50^b^	96.20^b^	69.00^a^	5.19	5.99	<0.001	<0.001	<0.001
Proteobacteria	1.96^ab^	1.03^ab^	0.39^a^	2.55^b^	0.65^a^	1.36^ab^	2.44^b^	0.72	0.83	NS	NS	NS
Synergistetes	0.00	0.01	0.26	0.01	0.02	0.00	0.19	0.11	0.12	NS	NS	NS
Tenericutes	0.02	1.20	0.29	0.02	0.00	1.15	0.00	0.75	0.86	NS	NS	NS
Verrucomicrobia	0.00	0.00	0.07	0.00	0.05	0.00	0.00	0.04	0.05	NS	NS	NS

*Numbers displayed are percentage sequencing reads pertaining to that genus as a proportion of the total number of reads. Different superscripts are shown for values that differ significantly from each other (P < 0.05)*.

**Figure 3:**
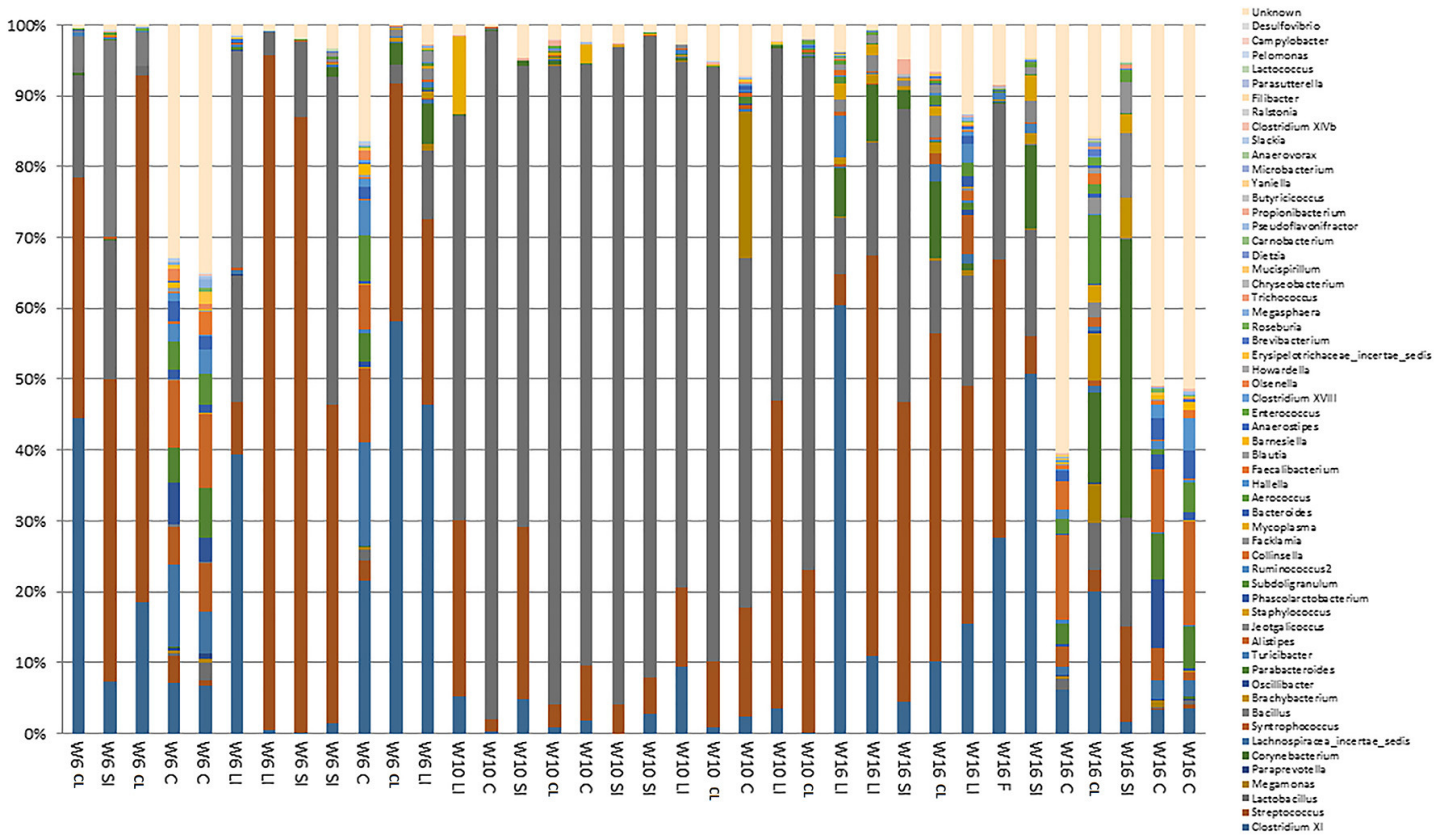
Proportional representation of the turkey gut microbiome on a genus level across the GI tract of birds at 6, 10, and 16 weeks of age. W, Week; SI, Small intestine; LI, Large intestine; C, Caeca; CL, Cloaca gut microbiome.

**Figure 4:**
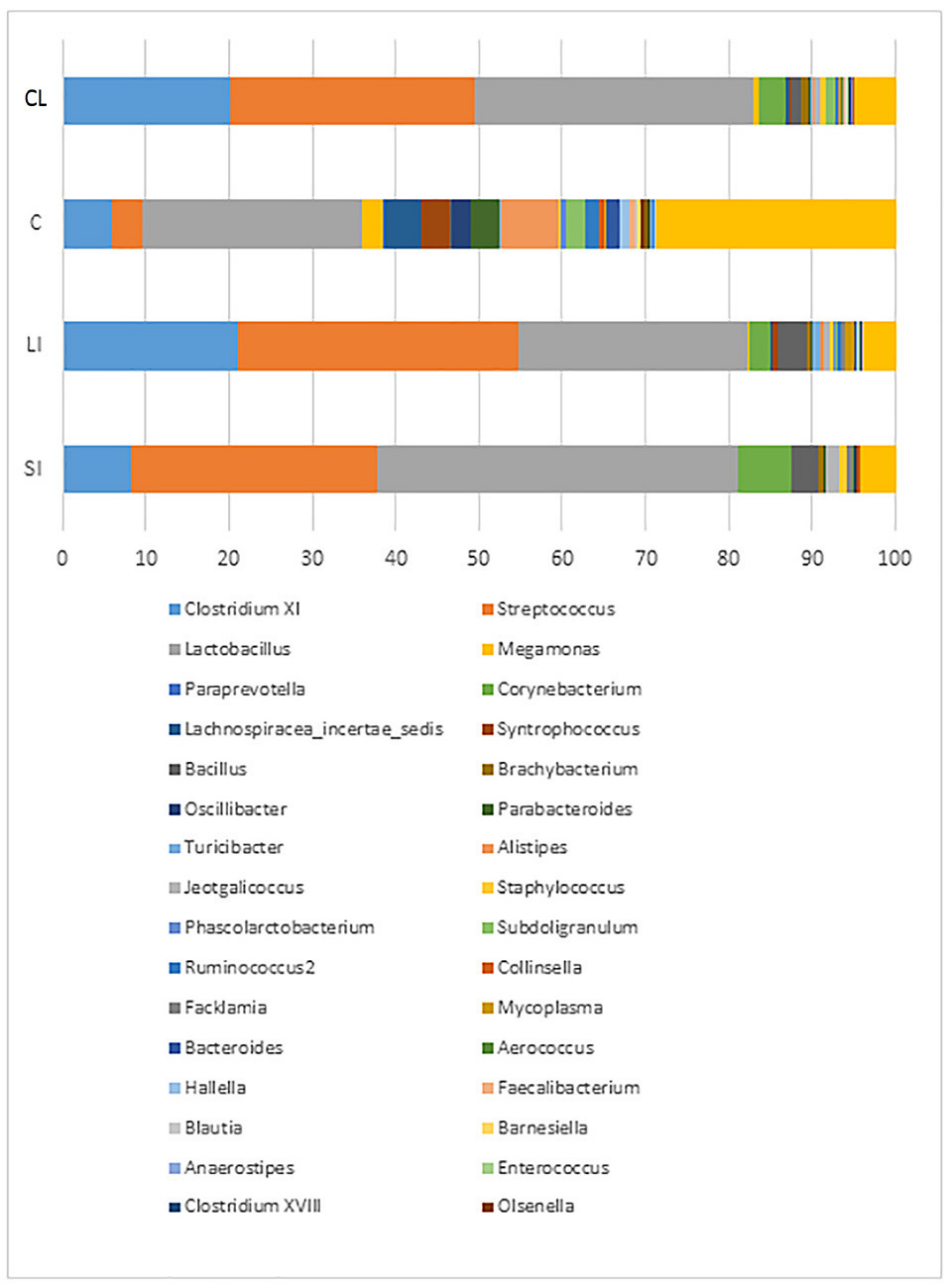
Proportional representation of the turkey gut microbiome on a genus level on average for all turkey ages combined and grouped by the GI tract location. SI, Small intestine; LI, Large intestine; C, Caeca; F, Cloaca gut microbiome.

On a family/genus level, and irrespective of GI tract location and bird age, *Lactobacillus, Streptococcus*, and *Clostridium XI* predominated (38.2, 28.1, and 13.0% respectively) (Figures 3, 4, Table 4, and Supplementary Tables 1–3). In terms of GI tract location, bacterial diversity in the small and large intestines were generally similar to each other in birds of all ages. The caecal bacterial diversity was highest in birds of all ages (Figures 3, 4, Table 4, Supplementary Figure 3 and Supplementary Tables 1–3). *Alistipes, Anaerovorax, Bacteroides, Barnesiella, Blautia, Butyricicoccus, Campylobacter, Clostridium XIVb, Hallela, Paraprevotella, Phascolarctobacterium, Pseudoflavonifractor, Roseburia, Ruminococcus, Slackia, Subdoligranulum, Syntrophococcus*, and unclassified bacteria were significantly (*P* < 0.05) higher in the caecum compared to the small and large intestine, whereas *Streptococcus* were significantly (*P* < 0.05) lower in abundance (Figures 3, 4, Table 4, and Supplementary Tables 1–3). Significant interactions between turkey age and GI tract location was seen for *Alistepes, Anaerovorax, Bacteroides, Barnsiella, Howardella, Megaspahaera, Olsenella, Parabacteroides, Pelomonas, Ruminococcus, Slackia, Subdoligranulum, Syntrophococcus* and unknown bacterial genera was seen (Table 4). The turkey cloacal microbiota showed most similarity to the microbiota within the large intestine, which is perhaps understandable given their close proximity (Figures 3, 4, Table 4, and Supplementary Tables 1–3). When considering the effect of turkey development on the GI tract microbiome, it is apparent that 10 week old birds show the most difference in their GI tract microbiome as a whole when compared to 6 and 16 week old birds (Figures 3, 4, Table 4, Supplementary Tables 1–3 and Supplementary Figure 3). Ten-week-old turkeys commonly showed less diversity based on alpha diversity indices, when compared with 6 and 16 week old birds (Supplementary Figure 3). Ten week old turkeys had less *Alistipes, Jeotgalicoccus, Parabacteroides, Phascolarctobacterium*, and *Streptococcus* and more *Campylobacter* and *Lactobacillus* than 6 and 16 week old birds. Sixteen week old birds also had more *Clostridium XI, Corynebacterium, Facklamia* and unclassified bacteria than 6 and 10 week old birds (Figures 3, 4, Table 3, and Supplementary Tables 1–3). Estimates of beta diversity and dispersion suggested that the highly significant differences in diversity observed between age and location groups (*P* < 0.001) are not due to the variation in homogeneity between the groups (*P* > 0.1). Across the entire dataset, significant (*P* < 0.001 and *P* < 0.01), moderate (*r* = 0.67 and *r* = 0.49) correlations were seen between the presence/abundance of *Campylobacter* with *Megamonas* and *Lactobacillus* at both genus and OTU level.

**Table 4:** Bacterial genera present within the small intestine, caecum, large intestine, and cloaca of mature turkeys.

**Bacterial genus**	**Sample location**				**Age**			**SED**		***P***		
	**SI**	**LI**	**C**	**F**	**6**	**10**	**16**	**Age**	**Sample**	**Age**	**Sample**	Age^*^Gut
*Aerococcus*	0.26	0.11	0.01	0.21	2.70	2.20	3.89	0.12	0.14	0.010	NS	NS
*Alistipes*	0.06^a^	0.31^a^	6.92^b^	0.25^a^	2.28^b^	0.11^a^	3.27^b^	1.00	1.16	0.010	<0.001	<0.001
*Anaerostipes*	0.00	0.04	0.08	0.05	0.02^a^	0.00^a^	0.11^b^	0.04	0.05	0.020	NS	NS
*Anaerovorax*	0.00^a^	0.01^a^	0.03^b^	0.04^a^	0.0^ab^	0.00^a^	0.02^b^	0.01	0.01	0.01	<0.001	0.031
*Bacillus*	3.10	3.40	0.10	1.10	5.70^a^	0.00^a^	0.00^ab^	2.68	3.10	NS	NS	NS
*Bacteroides*	0.03^a^	0.16^a^	1.70^b^	0.12^a^	0.55^ab^	0.06^a^	0.89^b^	0.27	0.31	0.020	<0.001	0.001
*Barnesiella*	0.00^a^	0.02^a^	0.50^b^	0.02^a^	0.23^a^	0.01^a^	0.16^a^	0.11	0.13	NS	<0.001	0.055
*Blautia*	0.00^a^	0.03^a^	0.18^b^	0.11^ab^	0.09^ab^	0.01^a^	0.14^b^	0.06	0.07	NS	NS	NS
*Brachybacterium*	0.87	0.38	0.03	1.00	0.15^a^	0.04^a^	1.53^b^	0.53	0.61	0.014	NS	NS
*Brevibacterium*	0.07	0.05	0.00	0.11	0.02^ab^	0.00^a^	0.14^b^	0.06	0.07	NS	NS	NS
*Butyricicoccus*	0.00^a^	0.00^a^	0.02^b^	0.00^a^	0.01^a^	0.00^a^	0.01^a^	0.01	0.01	NS	0.03	NS
*Campylobacter*	0.00^a^	0.01^a^	0.03^b^	0.03^b^	0.00^a^	0.05^b^	0.00^a^	0.02	0.03	0.050	NS	NS
*Carnobacterium*	0.02	0.01	0.00	0.05	0.00	0.00	0.05	0.03	0.03	NS	NS	NS
*Chryseobacterium*	0.03	0.03	0.00	0.04	0.02^a^	0.00^a^	0.06^a^	0.03	0.04	NS	NS	NS
*Clostridium_XI*	8.10	21.20	5.90	20.10	2.10^b^	2.60^a^	17.80^b^	6.33	7.31	0.020	NS	NS
*Clostridium_XVIII*	0.00	0.02	0.06	0.02	0.03^a^	0.00^a^	0.05^a^	0.03	0.03	NS	NS	NS
*Clostridium_XlVb*	0.00	0.01	0.05^b^	0.01	0.01^ab^	0.00^a^	0.04^b^	0.01	0.02	0.044	0.025	NS
*ColliNSella*	0.01	0.10	0.56	0.01	0.06	0.01	0.44	0.28	0.32	NS	NS	NS
*Corynebacterium*	6.20	2.50	0.10	3.10	0.90^a^	0.20^a^	7.80^b^	2.58	2.98	0.01	NS	NS
*Enterococcus*	0.05^a^	0.07^a^	0.03^a^	0.30^b^	0.07	0.10	0.16	0.07	0.08	NS	0.009	NS
*Facklamia*	0.66	0.40	0.00	0.39	0.19^a^	0.02^a^	0.88^b^	0.32	0.37	0.029	NS	NS
*Hallella*	0.00^a^	0.10^a^	1.01^b^	0.04^a^	0.22	0.04	0.59	0.29	0.34	NS	0.013	NS
*Howardella*	0.00	0.01	0.04	0.02	0.03	0.00	0.02	0.01	0.02	NS	NS	0.003
*Jeotgalicoccus*	1.50	0.72	0.02	0.70	0.26^a^	0.05^a^	1.89^b^	0.60	0.70	0.009	NS	NS
*Lactobacillus*	43.40^b^	27.50^ab^	26.40^a^	33.50^b^	10.8^a^	74.7^b^	12.6^a^	5.16	5.96	<0.001	0.029	NS
*Lactococcus*	0.05	0.02	0.00	0.00	0.04^b^	0.00^a^	0.02^ab^	0.02	0.02	NS	NS	NS
*Megamonas*	0.02	0.24	2.57	0.70	0.17	1.76	0.71	1.45	1.67	NS	NS	NS
*Megasphaera*	0.00	0.06	0.28	0.02	0.17	0.02	0.09	0.08	0.09	NS	0.013	0.053
*Microbacterium*	0.00	0.00	0.00	0.02	0.00	0.00	0.03	0.01	0.02	NS	NS	NS
*Mucispirillum*	0.00	0.00	0.05	0.00	0.00	0.00	0.04	0.03	0.03	NS	NS	NS
*Mycoplasma*	0.02	1.19	0.29	0.02	0.00	1.14	0.00	0.74	0.85	NS	NS	NS
*Olsenella*	0.01	0.02	0.44	0.01	0.32^b^	0.02^a^	0.03^a^	0.12	0.14	0.022	0.006	<0.001
*Oscillibacter*	0.02	0.10	2.32	0.06	0.89	0.03	0.96	0.70	0.80	NS	0.017	NS
*Parabacteroides*	0.03	0.24	3.51	0.08	1.39^b^	0.12^a^	1.38^b^	0.51	0.59	0.027	<0.001	<0.001
*Paraprevotella*	0.00^a^	0.01^a^	0.21^b^	0.01^a^	0.12	0.02	0.05	0.06	0.06	NS	0.006	NS
*Parasutterella*	0.00	0.01	0.06	0.01	0.01	0.03	0.01	0.02	0.03	NS	NS	NS
*Pelomonas*	0.05^b^	0.01^a^	0.00^a^	0.01^a^	0.03^b^	0.02^ab^	0.00^a^	0.01	0.02	NS	0.023	0.023
*Phascolarctobacterium*	0.02^a^	0.17^a^	0.75^b^	0.04^a^	0.29^ab^	0.02^a^	0.43^b^	0.17	0.19	NS	0.002	NS
*Propionibacterium*	0.32	0.05	0.02	0.15	0.03	0.16	0.22	1.47	0.17	NS	NS	NS
*Pseudoflavonifractor*	0.00^a^	0.00^a^	0.09^b^	0.01^a^	0.03	0.00	0.05	0.03	0.04	NS	0.045	NS
*Roseburia*	0.00^a^	0.02^a^	0.15^b^	0.00^a^	0.06	0.00	0.07	0.05	0.06	NS	0.038	NS
*Ruminococcus*	0.02^a^	0.33^a^	1.54^b^	0.11^a^	0.93	0.04	0.53	0.38	0.43	NS	0.005	<0.001
*Slackia*	0.00^a^	0.01^a^	0.13^b^	0.01^a^	0.09	0.00	0.02	0.03	0.04	0.031	0.004	<0.001
*Staphylococcus*	0.81	0.50	0.02	0.54	0.08	0.09	1.22	0.29	0.34	<0.001	NS	NS
*Streptococcus*	29.60^b^	33.50^b^	3.70^a^	29.40^b^	37.60^b^	14.20^a^	20.30^ab^	08.66	0.10	0.031	0.021	NS
*Subdoligranulum*	0.06^a^	0.40^a^	2.43^b^	1.27^ab^	1.30^ab^	0.03^a^	1.78^b^	0.78	0.90	NS	NS	0.038
*Syntrophococcus*	0.05^a^	0.73^a^	3.53^b^	0.27^a^	1.94^b^	0.06^a^	1.43^ab^	0.77	0.89	0.055	0.001	<0.001
*Trichococcus*	0.06	0.02	0.00	0.05	0.00^a^	0.00^a^	0.10^b^	0.04	0.05	NS	NS	NS
*Turicibacter*	0.15	0.89	0.19	0.29	0.24	0.05	0.85	0.40	0.45	NS	NS	NS
*Unknown*	3.20^a^	3.20^a^	28.50^b^	4.50^a^	8.00^a^	2.80^a^	18.70^b^	4.03	4.65	0.001	<0.001	<0.001
*Yaniella*	0.01^a^	0.00^a^	0.00^a^	0.02^a^	0.00^a^	0.00^ab^	0.02^a^	0.01	0.01	NS	NS	NS

*Numbers displayed are percentage sequencing reads pertaining to that genus as a proportion of the total number of reads. Different superscripts are shown for values that differ significantly from each other (P < 0.05)*.

### Turkey microbiome function

**Figure 5:**
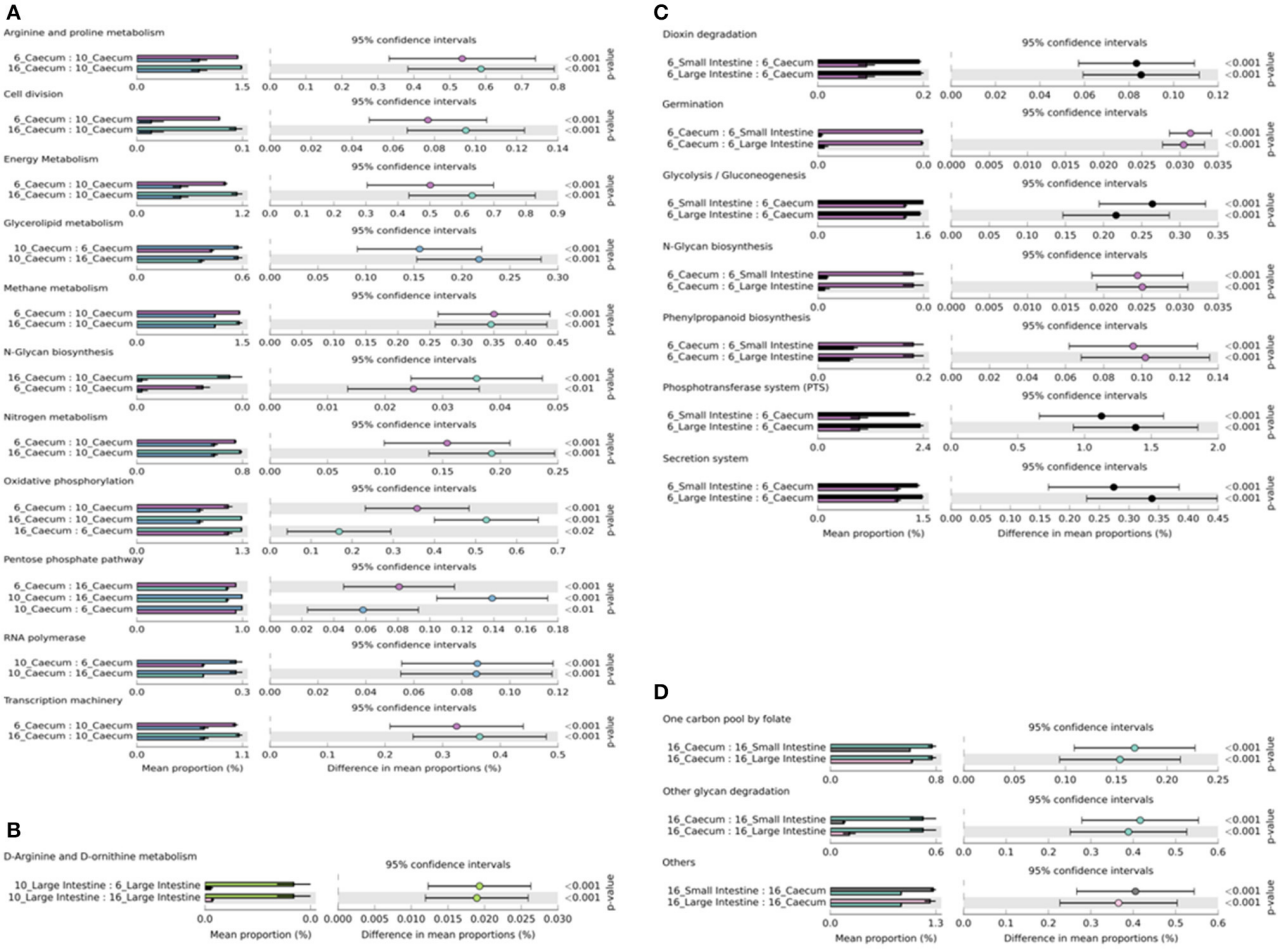
PICRUSt predicted metagenomes show significant differences in functionality in birds of different ages and GI tract locations. **(A)** Displays significant comparisons of *Post-Hoc* mean proportions and differences in mean proportions of KEGG pathway representation in the caeca of 6, 10, and 16 week birds. **(B)** Shows significant comparisons in the Large intestine of 6, 10, and 16 week birds. **(C)** Represents significant comparisons between caecal, small intestinal, and large intestine functionality in 6 week old birds. **(D)** Shows significant comparisons between GI tract locations in 16 week old birds.

PICRUSt data illustrated a range of potential functionalities (Supplementary Figure 1). ANOVA of PICRUSt data showed significant differences (<0.05) in 11 KEGG pathways (Arginine and proline metabolism, cell division, energy metabolism, glycerolipid metabolism, methane metabolism, N-glycan biosynthesis, nitrogen metabolism, oxidative phosphorylation, pentose phosphate pathway, and transcription machinery) in the caecal microbiome when comparing 6 to 10 and 10 to 16 week old birds (all least abundant within 10 week old birds except RNA polymerase and transcription machinery which were at their lowest abundance in 6 week old birds), and two of these pathways (pentose phosphate and oxidative phosphorylation) also differed between 6 and 16 week old birds (pentose phosphate pathway was significantly higher in abundance within 6 week old birds compared with 16 week old birds and vice versa for gene abundances correlating to oxidative phosphorylation; Figure 5A). In the large intestine the D-arginine and the D-ornithine pathway were significantly different in abundance when comparing 6 to 10 and 10 to 16 week old birds (highest abundances found in 10 week old birds; Figure 5B). In 6 week old birds metagenomic function differed when comparing the caeca to the large intestine and the caeca to the small intestine in 7 KEGG pathways (dioxin degradation, germination, N-glycan biosynthesis and phenylpropanoid biosynthesis were higher in the caeca whereas Glycolysis/gluconeogenesis, phosphotransferase system (PTS) and secretion system gene abundances were lower in the caeca of 6 week old birds; Figure 5C), no significant differences were seen between the small and large intestines. In 16 week old birds comparing the caeca to the large intestine and the caeca to the small intestine highlighted 3 KEGG pathways significantly different (one carbon pool by folate was higher in the caeca, whereas gene abundances for other glycan degradation and others were higher in the SI of 16 week old birds; Figure 5D), again no differences were seen between the small and large intestines. Ten week old birds showed no significant differences in functionality along the GI tract. Principal coordinate analysis (PCA) of PC1 against PC2 showed some distinction in the function of the bacteria within 10 week old birds. In total PC1 accounts for 48.1% of the variability between samples and PC2 accounts for 31.4% (Supplementary Figure 2).

### Total bacterial and *Campylobacter* spp. 16S rDNA quantitation

Total bacterial 16S rDNA concentrations were higher in the caeca, with the highest concentrations found in the caeca of 16 week old birds (Table 5). *Campylobacter* spp. 16S rDNA concentrations were also higher in the caecum, with the highest concentrations found in the caeca of 10 week old birds (Table 5).

**Table 5:** Effects of gastrointestinal tract location and turkey age on total bacterial and *Campylobacter* spp. 16S rDNA concentration.

	**Sample location**				**Age**			**SED**		***P***		
	**SI**	**LI**	**C**	**F**	**6**	**10**	**16**	**Age**	**Sample**	**Age**	**Sample**	Age^*^Gut
Total bacteria	9.50^a^	11.80^a^	43.70^b^	6.20^a^	5.50^a^	10.00^a^	37.90^b^	3.72	4.30	<0.001	<0.001	<0.001
*Campylobacter* spp.	2.64^a^	3.42^b^	4.56^c^	3.56^b^	2.88^a^	5.71^b^	2.04^c^	0.30	0.34	<0.001	<0.001	<0.01

*Numbers shown are log_10_ ng/mg sample dry matter. Different superscripts are shown for values that differ significantly from each other (P < 0.05)*.

## Discussion

In this study we characterized the microbiome across the GI tract of maturing turkeys, with a specific focus on *Campylobacter* spp. Many studies have been completed on the microbiome of chickens but fewer exist with respect to the turkey microbiome. We show that both GI tract location and turkey age have a significant effect on the whole gut microbiome present. We also show that *Campylobacter* spp. 16S rDNA concentrations are most abundant within the caeca of 10 week old birds compared with 6 and 16 week old birds (Table 6). Understanding the turkey microbiome in various locations of the GI tract and over turkey maturation is crucial in order to understand production efficiency and also the pathogen load and risk with respect to human consumption.

**Table 6:** Total bacterial and *Campylobacter* spp. 16S rDNA concentration in the small intestine (SI), large intestine (LI), caecum (C), and feces (f) of growing turkeys.

	**Sample location**				**SED**	***P***
	**SI**	**LI**	**C**	**F**		
**TOTAL BACTERIA**						
6 weeks	2.9^a^	3.1^a^	12.3^b^	2.6^c^	5.70	<0.001
10 weeks	8.2^ab^	13.0^ab^	20.3^b^	4.4^a^		
16 weeks	15.0^a^	18.3^a^	97.5^b^	12.7^a^		
***Campylobacter*** **spp**.						
6 weeks	2.3^a^	2.5^ab^	3.8^c^	3.0^b^	0.57	<0.001
10 weeks	3.7^a^	5.5^ab^	7.6^c^	6.6^bc^		
16 weeks	2.2^a^	2.2^a^	2.4^a^	1.6^a^		

*Numbers shown are log_10_ ng/mg sample dry matter. Different superscripts are shown for values that differ significantly from each other (P < 0.05)*.

On a phylum level, Firmicutes, Bacteroidetes, Actinobacteria, and Proteobacteria dominated within the microbiomes of the turkeys across age and GI tract location. This is in line with previously published metataxonomic data for the chicken and turkey gut microbiomes (Qu et al., 2008; Yeoman et al., 2012; Oakley et al., 2013; Wei et al., 2013; Choi et al., 2014; Mohd et al., 2015; Molina-Borda et al., 2016). In terms of GI tract location, Bacteroidetes were significantly more abundant in the caeca, whilst the converse was true for Firmicutes, which were higher in abundance in the small and large intestine, and in cloaca material compared with caecal abundances. Proteobacteria predominated in the cloaca area, which is perhaps unsurprising as they are more tolerant of oxygen, which is likely to penetrate the cloaca and therefore be higher in abundance. Similar data have also been reported for the spatial nature of the broiler chicken microbiome (Choi et al., 2014; Mohd et al., 2015). When assessing the effect of age, on average Actinobacteria, Bacteroidetes, and Proteobacteria were more abundant in the GI tracts of 16 week old turkeys, whilst Firmicutes were higher in abundance in the GI tract of 6 and 10 week birds compared with 16 week birds.

On a genus/family level *Lactobacillus, Streptococcus*, and *Clostridium_XI*, dominated irrespective of gut location and turkey age. This data is in line with other reported data investigating the poultry gut microbiome to test differing hypotheses (Danzeisen et al., 2013, 2015; Choi et al., 2014; Videnska et al., 2014; Mohd et al., 2015; Oakley and Kogut, 2016). In terms of GI tract location, *Alistipes, Bacteroides, Barnesiella, Butyricoccus, Clostridium_XIVb, Hallela, Paraprevotella, Phascolarctobacterium, Pseudoflavonifractor, Roseburia, Ruminococcus, Slackia, Syntrophococcus* were higher in abundance in the caeca irrespective of turkey age. *Blautia* and *Campylobacter* had higher abundances in the caeca and cloaca than within the small and large intestines, whilst *Anaerovorax* and *Corynebacterium* dominated in the cloaca. *Lactobacillus* and *Streptococcus* had a significantly lower abundance in the caeca compared to abundances in the other GI tract locations. When assessing the effect of age, 10 week old birds generally showed the greatest difference in their GI tract microbiome as a whole when compared to 6 and 16 week old birds. Ten week old turkeys generally had less *Alistipes, Jeotgalicoccus, Parabacteroides, Phascolarctobacterium*, and *Streptococcus* and more *Campylobacter* and *Lactobacillus* than 6 and 16 week old birds. Sixteen week old birds also had more *Clostridium XI, Corynebacterium, Facklamia* and unclassified bacteria than 6 and 10 week old birds. Danzeisen et al. (2015) in a study investigating the ileal and caecal microbiome during maturation in 45 turkeys also noted that *Clostridium XI* increased in abundance in the GI tract as the birds aged, possibly as a consequence of their ability to ferment aromatic amino acids. Danzeisen et al. (2013) also noted that age was a key factor affecting the microbiome with *Lactobacillus* increasing in the ilea of turkeys as they age. We also found an increase in *Lactobacillus* in the SI in 10 week old birds compared with 6 week old birds, but numbers decreased within 16 week old birds. Alpha diversity indices showed that the caecal microbiome of 6 and 16 week old birds were higher in diversity compared with 10 week old birds.

Sequencing the rRNA gene of a gut microbiome is relatively simple and cost-effective, nonetheless understanding the function of the microbiome is key for understanding interrelationships with the host. Inferring function based on diversity of bacteria present can be difficult as the bacteria often transfer genes, and show a high degree of reliance and redundancy (Allison and Martiny, 2008). The relatively recently developed PICRUSt program has proved to be effective at obtaining functional predictions from 16S rRNA taxonomy data (Langille et al., 2013). Therefore, in an attempt to gain functional insight into the spatial and temporal function of the turkey gut microbiome we used PICRUSt. The main observations from the PICRUSt function data were that 10 week old birds differed significantly in the function of their caecal microbiome compared to birds of 6 and 16 weeks of age.

In this study we also show using next generation sequencing and QPCR that the abundance of thermophilic *Campylobacter* spp. in the turkey GI tract is at its highest within the caeca of 10 week old birds. It should also be noted that *Campylobacter* were underrepresented in our sequencing data compared to our QPCR data, despite the sequencing primers having a 100% match to *Campylobacter* spp. Therefore, the reason for this cannot be determined although it is possibly a consequence of targeting secondary DNA structures in *Campylobacter*. Irrespective, in a previous study using pre-next generation sequencing technology, Scupham (2009) suggested that *Campylobacter* spp. vary with turkey age and are often linked with transition points within the whole microbiome diversity. Also, Thibodeau et al. (2015) suggested that levels of *Campylobacter* in the caeca of chickens were associated with changes in the microbiome particularly increasing in abundance when increases in *Bifidobacterium, Mollicutes*, and *Clostria* are seen. We saw a correlation with a rise in *Campylobacter* spp. abundance in the caeca and increases in *Lactobacillus* and *Megamonas*, whether they are causative linkages would need further investigation. Data for broiler chickens also suggests that post 8 weeks of age, the abundance of *Campylobacter* spp. in the caeca is reduced, which is hypothesized to be due to acquired immunity (Achen et al., 1998; Newell and Fearnley, 2003; Humphrey et al., 2014; Wigley, 2015; Reid et al., 2016). This raises the question that *Campylobacter* spp. are not natural commensals of the chicken gut, and also suggests that slaughtering at a later stage is potentially beneficial for human health. Our data for the turkey caecal microbiome also suggests that as turkeys reach slaughter age, acquired immunity may play a role in suppressing *Campylobacter* spp., although this hypothesis needs testing. These data highlight the complex interactions of the microbiome and the need to study the whole microbiome, and not the pathogens themselves in isolation.

In summary, in this study we show that the turkey gut microbiome, across the GI tract, changes in terms of taxonomy, diversity and function as the turkey matures with the main changes occuring in the caeca. We also show that *Campylobacter* reside predominantly in the caeca and numbers are higher at 10 weeks of age with reductions seen at age of slaughter. This study provides an understanding of the turkey gut microbiome, and contributes to the low number of publications available within the field as compared with chicken GI tract data. Understanding the microbial ecology of the turkey gastrointestinal tract is essential in terms of understanding production efficiency and in order to develop novel strategies for targeting *Campylobacter* spp.

## Author contributions

Study idea and design: AC, JM, SB, CN, and SH; trial setup and sample collection HV, KW, and VT; sample analysis: TW, SC, LAO, LBO, and CG. Paper concept and writing: AC, TW, and SH. All authors discussed the results and commented on the manuscript at all stages.

## Ethics statement

The study was conducted under a home office license.

### Conflict of interest statement

The authors declare that the research was conducted in the absence of any commercial or financial relationships that could be construed as a potential conflict of interest.
