# Supplementary material for: Characterization of the Microbiome along the Gastrointestinal Tract of Growing Turkeys
Source: Front Microbiol. 2017 Jun 22;8:1089. doi: 10.3389/fmicb.2017.01089 (PMC5479886; doi:10.3389/fmicb.2017.01089)
Supplement: Supplementary file 1 [file Table1.DOCX]

**Supplementary Table 1.** Bacterial genera present within the small intestine (SI), caecum (C), large intestine (LI), and Cloaca (CL) of 6 week old turkeys. Numbers displayed are percentage sequencing reads pertaining to that genus as a proportion of the total number of reads.

| **Bacterial genus** | **Sample location** | | | | **SED** | ***P*** |
| --- | --- | --- | --- | --- | --- | --- |
|  | SI LI C CL | | | |  |  |
| *Aerococcus* | 0.04 | 0.04 | 0.01 | 0.02 | 0.03  0.92  0.02  0.00  9.81  0.29  0.27  0.06  0.26  0.05  0.01  ND  0.01  0.03  13.56  0.07  0.02  0.08  0.04  1.55  0.08  0.38  0.22  0.02  0.44  8.65  0.06  0.02  0.19  ND  0.00  ND  0.21  0.10  0.65  0.17  0.02  0.03  0.23  0.06  ND  0.12  0.53  0.04  0.07  2.35  0.57  1.06  0.00  0.22  4.23  ND | NS  <0.001  NS  <0.001  NS  <0.001  0.029  <0.001  NS  NS  NS  ND  NS  NS  NS  NS  NS  NS  NS  NS  NS  NS  0.011  0.002  NS  NS  NS  NS  0.034  ND  NS  ND  <0.001  0.022  <0.001  NS  NS  0.025  0.005  NS  ND  NS  <0.001  <0.001  NS  NS  <0.001  <0.001  NS  NS  <0.001  ND |
| *Alistipes* | 0.12^a^ | 0.20^a^ | 8.76^b^ | 0.04^a^ |  |  |
| *Anaerostipes* | 0.00 | 0.06 | 0.08 | 0.00 |  |  |
| *Anaerovorax* | 0.00^a^ | 0.00^a^ | 0.04^b^ | 0.00^a^ |  |  |
| *Bacillus* | 9.26 | 10.21 | 0.17 | 3.24 |  |  |
| *Bacteroides* | 0.04^a^ | 0.08^a^ | 2.07^b^ | 0.02^a^ |  |  |
| *Barnesiella* | 0.00^a^ | 0.02^a^ | 0.89^b^ | 0.00^a^ |  |  |
| *Blautia* | 0.00^a^ | 0.01^a^ | 0.34^b^ | 0.00^a^ |  |  |
| *Brachybacterium* | 0.04 | 0.34 | 0.03 | 0.21 |  |  |
| *Brevibacterium* | 0.04 | 0.06 | 0.00 | 0.00 |  |  |
| *Butyricicoccus* | 0.00 | 0.00 | 0.03 | 0.00 |  |  |
| *Campylobacter* | ND | ND | ND | ND |  |  |
| *Carnobacterium* | 0.00 | 0.00 | 0.00 | 0.01 |  |  |
| *Chryseobacterium* | 0.00 | 0.06 | 0.00 | 0.00 |  |  |
| *Clostridium_XI* | 2.97 | 28.70 | 11.82 | 40.37 |  |  |
| *Clostridium_XVIII* | 0.00 | 0.00 | 0.11 | 0.00 |  |  |
| *Clostridium_XlVb* | 0.00 | 0.00 | 0.03 | 0.00 |  |  |
| *Collinsella* | 0.00 | 0.00 | 0.24 | 0.00 |  |  |
| *Corynebacterium* | 0.54 | 2.00 | 0.07 | 1.11 |  |  |
| *Enterococcus* | 0.05 | 0.03 | 0.04 | 0.15 |  |  |
| *Facklamia* | 0.18 | 0.51 | 0.00 | 0.06 |  |  |
| *Hallella* | 0.00^a^ | 0.01^a^ | 0.87^b^ | 0.00^a^ |  |  |
| *Howardella* | 0.00^a^ | 0.00^a^ | 0.10^b^ | 0.00^a^ |  |  |
| *Jeotgalicoccus* | 0.17 | 0.53 | 0.02 | 0.33 |  |  |
| *Lactobacillus* | 25.40 | 10.26 | 1.44 | 6.15 |  |  |
| *Lactococcus* | 0.15 | 0.03 | 0.00 | 0.00 |  |  |
| *Megamonas* | 0.00 | 0.28 | 0.41 | 0.02 |  |  |
| *Megasphaera* | 0.00^a^ | 0.00^a^ | 0.60^b^ | 0.06^a^ |  |  |
| *Microbacterium* | ND | ND | ND | ND |  |  |
| *Mucispirillum* | 0.00 | 0.00 | 0.01 | 0.00 |  |  |
| *Mycoplasma* | ND | ND | ND | ND |  |  |
| *Olsenella* | 0.03^a^ | 0.04^a^ | 1.21^b^ | 0.00^a^ |  |  |
| *Oscillibacter* | 0.04^a^ | 0.10^a^ | 3.40^b^ | 0.00^a^ |  |  |
| *Parabacteroides* | 0.05^a^ | 0.29^a^ | 5.21^b^ | 0.03^a^ |  |  |
| *Paraprevotella* | 0.00 | 0.02 | 0.44 | 0.00 |  |  |
| *Parasutterella* | 0.00 | 0.00 | 0.03 | 0.00 |  |  |
| *Pelomonas* | 0.11^b^ | 0.02^a^ | 0.00^a^ | 0.00^a^ |  |  |
| *Phascolarctobacterium* | 0.05^a^ | 0.06^a^ | 1.04^b^ | 0.00^a^ |  |  |
| *Propionibacterium* | 0.00 | 0.00 | 0.10 | 0.00 |  |  |
| *Pseudoflavonifractor* | ND | ND | ND | ND |  |  |
| *Roseburia* | 0.00 | 0.00 | 0.24 | 0.00 |  |  |
| *Ruminococcus* | 0.00^a^ | 0.09^a^ | 3.60^b^ | 0.00^a^ |  |  |
| *Slackia* | 0.00^a^ | 0.00^a^ | 0.34^b^ | 0.00^a^ |  |  |
| *Staphylococcus* | 0.09 | 0.13 | 0.02 | 0.10 |  |  |
| *Streptococcus* | 57.99 | 42.87 | 2.46 | 47.14 |  |  |
| *Subdoligranulum* | 0.14^a^ | 0.15^a^ | 4.90^b^ | 0.02^a^ |  |  |
| *Syntrophococcus* | 0.11^a^ | 0.16^a^ | 7.49^b^ | 0.00^a^ |  |  |
| *Trichococcus* | 0.00 | 0.01 | 0.00 | 0.00 |  |  |
| *Turicibacter* | 0.03 | 0.34 | 0.25 | 0.33 |  |  |
| *Unknown* | 1.98^a^ | 1.69^a^ | 28.11^b^ | 0.32^a^ |  |  |
| *Yaniella* | ND | ND | ND | ND |  |  |
