# Supplementary material for: Characterization of the Microbiome along the Gastrointestinal Tract of Growing Turkeys
Source: Front Microbiol. 2017 Jun 22;8:1089. doi: 10.3389/fmicb.2017.01089 (PMC5479886; doi:10.3389/fmicb.2017.01089)
Supplement: Supplementary file 2 [file Table2.DOCX]

**Supplementary Table 2.** Bacterial genera present within the small intestine, caecum, large intestine, and cloaca of 10 week old turkeys. Numbers displayed are percentage sequencing reads pertaining to that genus as a proportion of the total number of reads.

| **Bacterial genus** | **Sample location** | | | | **SED** | ***P*** |
| --- | --- | --- | --- | --- | --- | --- |
|  | SI LI C CL | | | |  |  |
| *Aerococcus* | 0.05 | 0.01 | 0.00 | 0.02 | 0.02  0.16  ND  ND  0.05  0.13  0.02  0.02  ND  ND  ND  0.08  ND  0.01  1.59  ND  0.00  0.01  0.21  0.13  0.05  0.05  0.01  0.08  13.48  ND  0.05  0.05  ND  ND  2.61  0.03  0.04  0.22  0.03  0.07  0.02  0.04  0.16  ND  0.00  0.05  0.01  0.13  0.09  0.03  0.13  ND  0.13  1.77  ND | NS  NS  ND  ND  NS  NS  NS  NS  NS  NS  NS  NS  ND  NS  0.046  ND  NS  NS  NS  NS  NS  NS  NS  NS  NS  ND  NS  NS  ND  ND  NS  NS  NS  NS  NS  NS  NS  NS  NS  ND  NS  NS  NS  NS  NS  NS  NS  ND  NS  NS  ND |
| *Alistipes* | 0.00 | 0.08 | 0.23 | 0.14 |  |  |
| *Anaerostipes* | ND | ND | ND | ND |  |  |
| *Anaerovorax* | ND | ND | ND | ND |  |  |
| *Bacillus* | 0.00 | 0.00 | 0.07 | 0.02 |  |  |
| *Bacteroides* | 0.00 | 0.00 | 0.18 | 0.06 |  |  |
| *Barnesiella* | 0.00 | 0.00 | 0.03 | 0.00 |  |  |
| *Blautia* | 0.00 | 0.00 | 0.02 | 0.00 |  |  |
| *Brachybacterium* | ND | ND | ND | ND |  |  |
| *Brevibacterium* | ND | ND | ND | ND |  |  |
| *Butyricicoccus* | ND | ND | ND | ND |  |  |
| *Campylobacter* | 0.00 | 0.02 | 0.08 | 0.10 |  |  |
| *Carnobacterium* | ND | ND | ND | ND |  |  |
| *Chryseobacterium* | 0.00 | 0.01 | 0.00 | 0.00 |  |  |
| *Clostridium_XI* | 1.52^ab^ | 0.63^b^ | 5.91^a^ | 2.48^a^ |  |  |
| *Clostridium_XVIII* | ND | ND | ND | ND |  |  |
| *Clostridium_XlVb* | 0.00 | 0.00 | 0.01 | 0.00 |  |  |
| *Collinsella* | 0.00 | 0.00 | 0.01 | 0.02 |  |  |
| *Corynebacterium* | 0.30 | 0.21 | 0.01 | 0.26 |  |  |
| *Enterococcus* | 0.00 | 0.04 | 0.03 | 0.32 |  |  |
| *Facklamia* | 0.00 | 0.06 | 0.00 | 0.03 |  |  |
| *Hallella* | 0.00 | 0.01 | 0.03 | 0.10 |  |  |
| *Howardella* | 0.00 | 0.00 | 0.00 | 0.02 |  |  |
| *Jeotgalicoccus* | 0.00 | 0.14 | 0.00 | 0.04 |  |  |
| *Lactobacillus* | 76.9 | 81.3 | 59.2 | 81.3 |  |  |
| *Lactococcus* | ND | ND | ND | ND |  |  |
| *Megamonas* | 0.00 | 0.12 | 6.86 | 0.08 |  |  |
| *Megasphaera* | 0.00 | 0.00 | 0.08 | 0.00 |  |  |
| *Microbacterium* | ND | ND | ND | ND |  |  |
| *Mucispirillum* | ND | ND | ND | ND |  |  |
| *Mycoplasma* | 0.05 | 3.58 | 0.88 | 0.07 |  |  |
| *Olsenella* | 0.00 | 0.00 | 0.04 | 0.02 |  |  |
| *Oscillibacter* | 0.00 | 0.00 | 0.06 | 0.05 |  |  |
| *Parabacteroides* | 0.02 | 0.06 | 0.32 | 0.09 |  |  |
| *Paraprevotella* | 0.00 | 0.00 | 0.05 | 0.02 |  |  |
| *Parasutterella* | 0.00 | 0.00 | 0.12 | 0.00 |  |  |
| *Pelomonas* | 0.04 | 0.01 | 0.00 | 0.02 |  |  |
| *Phascolarctobacterium* | 0.00 | 0.00 | 0.07 | 0.00 |  |  |
| *Propionibacterium* | 0.20 | 0.03 | 0.03 | 0.36 |  |  |
| *Pseudoflavonifractor* | ND | ND | ND | ND |  |  |
| *Roseburia* | 0.00 | 0.00 | 0.01 | 0.00 |  |  |
| *Ruminococcus* | 0.02 | 0.00 | 0.03 | 0.11 |  |  |
| *Slackia* | 0.00 | 0.00 | 0.02 | 0.00 |  |  |
| *Staphylococcus* | 0.13 | 0.03 | 0.00 | 0.21 |  |  |
| *Streptococcus* | 10.84 | 26.17 | 8.24 | 11.75 |  |  |
| *Subdoligranulum* | 0.00 | 0.02 | 0.03 | 0.08 |  |  |
| *Syntrophococcus* | 0.02 | 0.00 | 0.20 | 0.03 |  |  |
| *Trichococcus* | ND | ND | ND | ND |  |  |
| *Turicibacter* | 0.00 | 0.18 | 0.01 | 0.00 |  |  |
| *Unknown* | 2.65 | 2.15 | 3.25 | 2.99 |  |  |
| *Yaniella* | ND | ND | ND | ND |  |  |
