# Supplementary material for: Characterization of the Microbiome along the Gastrointestinal Tract of Growing Turkeys
Source: Front Microbiol. 2017 Jun 22;8:1089. doi: 10.3389/fmicb.2017.01089 (PMC5479886; doi:10.3389/fmicb.2017.01089)
Supplement: Supplementary file 3 [file Table3.DOCX]

**Supplementary Table 3.** Bacterial genera present within the small intestine, caecum, large intestine, and cloaca of 16 week old turkeys. Numbers displayed are percentage sequencing reads pertaining to that genus as a proportion of the total number of reads.

| **Bacterial genus** | **Sample location** | | | | **SED** | ***P*** |
| --- | --- | --- | --- | --- | --- | --- |
|  | SI LI C CL | | | |  |  |
| *Aerococcus* | 0.68 | 0.28 | 0.01 | 0.58 | 0.41  1.19  0.14  0.02  0.03  0.61  0.19  0.17  1.80  0.21  0.01  ND  0.09  0.01  16.25  0.07  0.04  0.97  8.36  0.22  0.11  0.90  0.03  1.91  7.07  0.01  0.01  0.13  0.05  0.08  ND  0.02  2.20  0.82  0.03  0.01  0.00  0.49  0.48  0.09  0.13  0.62  0.02  0.94  16.24  2.27  1.44  0.16  1.39  3.89  0.03 | NS  <0.001  NS  NS  NS  0.006  NS  NS  NS  NS  NS  ND  NS  NS  NS  NS  NS  NS  NS  NS  NS  NS  NS  NS  NS  NS  NS  NS  NS  NS  ND  0.005  NS  <0.001  0.005  NS  NS  NS  NS  NS  NS  NS  NS  NS  NS  NS  NS  NS  NS  <0.001  NS |
| *Alistipes* | 0.07^a^ | 0.66^a^ | 11.78^b^ | 0.57^a^ |  |  |
| *Anaerostipes* | 0.00 | 0.12 | 0.17 | 0.14 |  |  |
| *Anaerovorax* | 0.00 | 0.01 | 0.05 | 0.01 |  |  |
| *Bacillus* | 0.06 | 0.00 | 0.00 | 0.03 |  |  |
| *Bacteroides* | 0.03^a^ | 0.39^a^ | 2.84^b^ | 0.29^a^ |  |  |
| *Barnesiella* | 0.00 | 0.04 | 0.57 | 0.04 |  |  |
| *Blautia* | 0.00 | 0.07 | 0.19 | 0.31 |  |  |
| *Brachybacterium* | 2.55 | 0.76 | 0.05 | 2.74 |  |  |
| *Brevibacterium* | 0.17 | 0.08 | 0.00 | 0.32 |  |  |
| *Butyricicoccus* | 0.00 | 0.00 | 0.01 | 0.00 |  |  |
| *Campylobacter* | ND | ND | ND | ND |  |  |
| *Carnobacterium* | 0.00 | 0.01 | 0.00 | 0.13 |  |  |
| *Chryseobacterium* | 0.10 | 0.00 | 0.00 | 0.12 |  |  |
| *Clostridium_XI* | 18.91 | 28.87 | 4.30 | 19.30 |  |  |
| *Clostridium_XVIII* | 0.00 | 0.07 | 0.07 | 0.07 |  |  |
| *Clostridium_XlVb* | 0.00 | 0.00 | 0.11 | 0.02 |  |  |
| *Collinsella* | 0.02 | 0.28 | 1.42 | 0.02 |  |  |
| *Corynebacterium* | 17.86 | 5.23 | 0.15 | 7.91 |  |  |
| *Enterococcus* | 0.09 | 0.12 | 0.00 | 0.44 |  |  |
| *Facklamia* | 0.02 | 0.20 | 0.22 | 0.07 |  |  |
| *Hallella* | 0.00 | 0.19 | 2.14 | 0.02 |  |  |
| *Howardella* | 0.00 | 0.01 | 0.01 | 0.00 |  |  |
| *Jeotgalicoccus* | 4.33 | 1.47 | 0.04 | 1.72 |  |  |
| *Lactobacillus* | 23.58 | 13.17 | 0.70 | 12.98 |  |  |
| *Lactococcus* | 0.02 | 0.02 | 0.00 | 0.01 |  |  |
| *Megamonas* | 0.05 | 0.31 | 0.45 | 2.00 |  |  |
| *Megasphaera* | 0.01 | 0.17 | 0.17 | 0.01 |  |  |
| *Microbacterium* | 0.03 | 0.00 | 0.00 | 0.07 |  |  |
| *Mucispirillum* | 0.00 | 0.00 | 0.15 | 0.00 |  |  |
| *Mycoplasma* | ND | ND | ND | ND |  |  |
| *Olsenella* | 0.00^a^ | 0.03^a^ | 0.08^b^ | 0.00^a^ |  |  |
| *Oscillibacter* | 0.01 | 0.21 | 3.50 | 0.13 |  |  |
| *Parabacteroides* | 0.03^a^ | 0.38^a^ | 5.00^b^ | 0.11^a^ |  |  |
| *Paraprevotella* | 0.00^a^ | 0.02^a^ | 0.15^b^ | 0.01^a^ |  |  |
| *Parasutterella* | 0.00 | 0.02 | 0.02 | 0.02 |  |  |
| *Pelomonas* | 0.00 | 0.00 | 0.00 | 0.01 |  |  |
| *Phascolarctobacterium* | 0.00 | 0.46 | 1.13 | 0.11 |  |  |
| *Propionibacterium* | 0.73 | 0.05 | 0.00 | 0.07 |  |  |
| *Pseudoflavonifractor* | 0.00 | 0.00 | 0.17 | 0.02 |  |  |
| *Roseburia* | 0.00 | 0.06 | 0.21 | 0.01 |  |  |
| *Ruminococcus* | 0.03 | 0.89 | 0.98 | 0.21 |  |  |
| *Slackia* | 0.00 | 0.01 | 0.02 | 0.03 |  |  |
| *Staphylococcus* | 2.20 | 1.33 | 0.04 | 1.32 |  |  |
| *Streptococcus* | 20.00 | 31.45 | 0.29 | 29.45 |  |  |
| *Subdoligranulum* | 0.05 | 1.02 | 2.35 | 3.70 |  |  |
| *Syntrophococcus* | 0.03 | 2.02 | 2.89 | 0.76 |  |  |
| *Trichococcus* | 0.18 | 0.04 | 0.00 | 0.16 |  |  |
| *Turicibacter* | 0.42 | 2.16 | 0.30 | 0.52 |  |  |
| *Unknown* | 4.90^a^ | 5.71^a^ | 54.10^b^ | 10.19^a^ |  |  |
| *Yaniella* | 0.02 | 0.00 | 0.00 | 0.05 |  |  |
