# Supplementary figures and images for: Characterization of the Microbiome along the Gastrointestinal Tract of Growing Turkeys
Source: Front Microbiol. 2017 Jun 22;8:1089. doi: 10.3389/fmicb.2017.01089 (PMC5479886; doi:10.3389/fmicb.2017.01089)

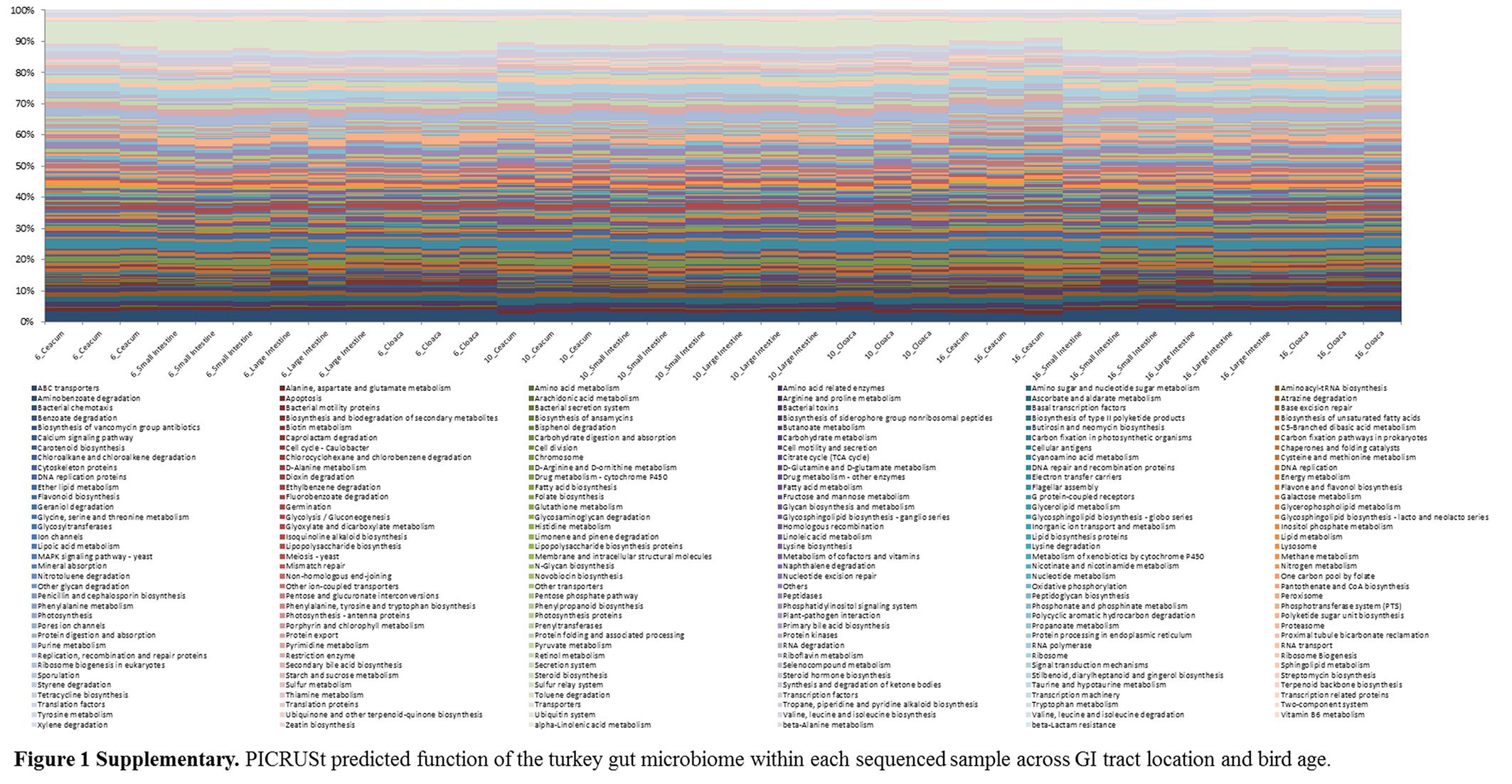

Supplement: Supplementary file 4 [file Image1.TIF]

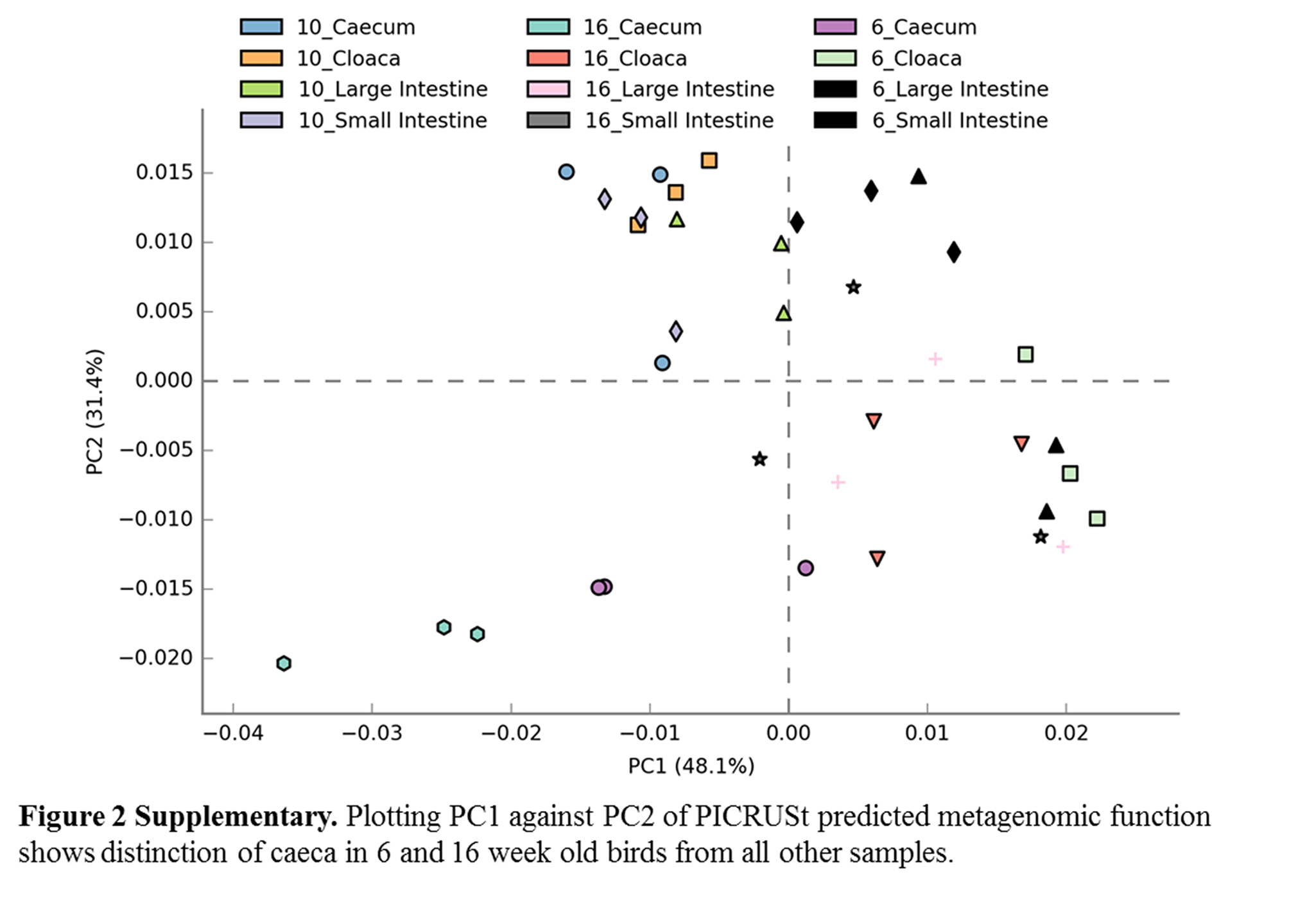

Supplement: Supplementary file 5 [file Image2.TIF]

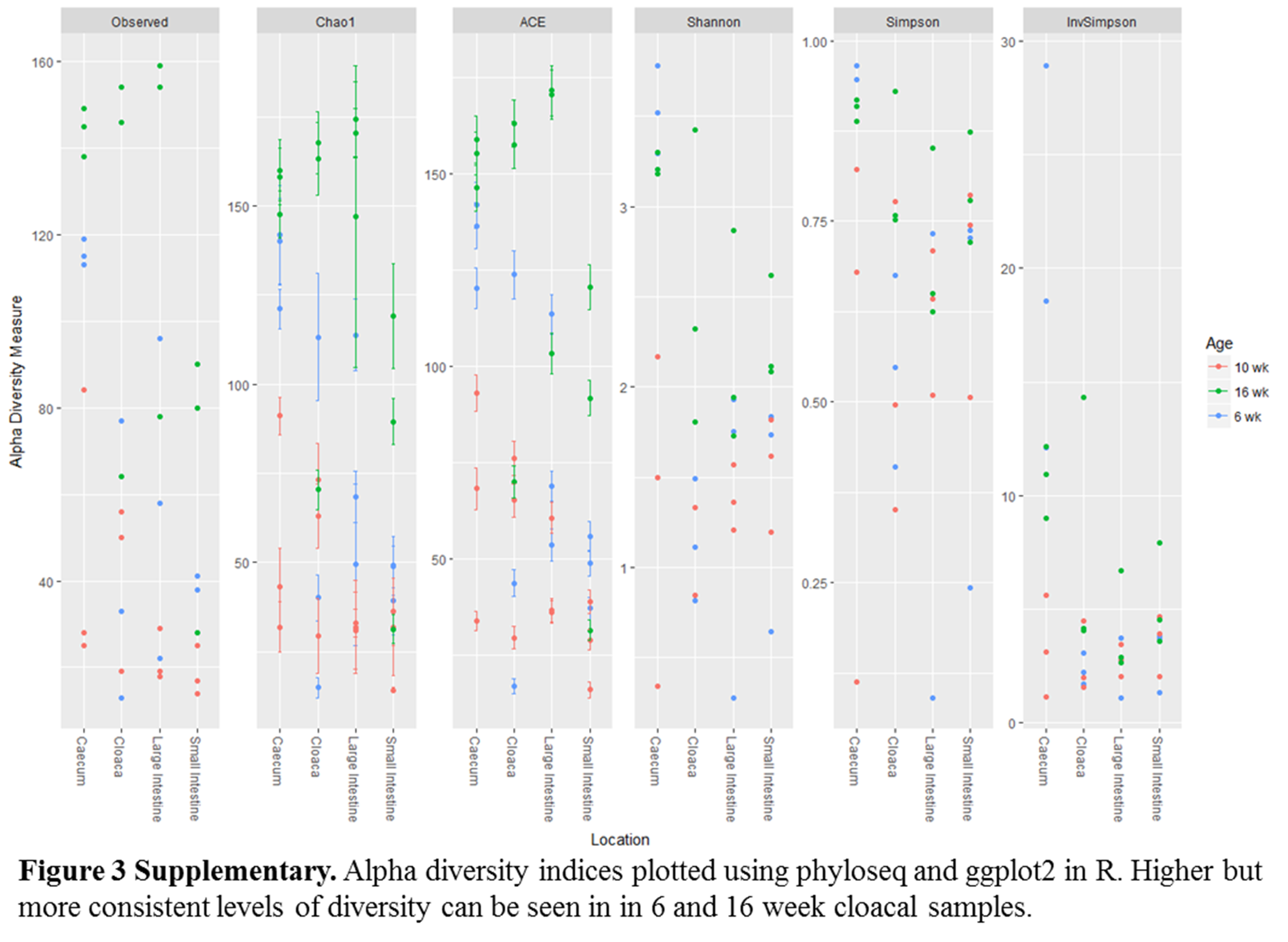

Supplement: Supplementary file 6 [file Image3.TIF]
